# Lower sertraline plasma concentration in patients co‐medicated with clozapine—Implications for pharmacological augmentation strategies in schizophrenia

**DOI:** 10.1002/prp2.1065

**Published:** 2023-02-24

**Authors:** Arnim Johannes Gaebler, Ekkehard Haen, Nagia Ben Omar, Katharina Endres, Christoph Hiemke, Georgios Schoretsanitis, Michael Paulzen

**Affiliations:** ^1^ Department of Psychiatry Psychotherapy and Psychosomatics, Medical Faculty Aachen Germany; ^2^ JARA ‐ Translational Brain Medicine Aachen Germany; ^3^ Institute of Physiology, Medical Faculty Aachen Germany; ^4^ Clinical Pharmacology Institute AGATE gGmbH Pentling Germany; ^5^ Department of Psychiatry and Psychotherapy, Clinical Pharmacology University of Regensburg Regensburg Germany; ^6^ Department of Pharmacology and Toxicology University of Regensburg Regensburg Germany; ^7^ Department of Psychiatry and Psychotherapy and Institute of Clinical Chemistry and Laboratory Medicine University Medical Center of Mainz Mainz Germany; ^8^ The Zucker Hillside Hospital, Psychiatry Research, Northwell Health New York New York USA; ^9^ University Hospital of Psychiatry Zurich Zurich Switzerland; ^10^ Alexianer Hospital Aachen Aachen Germany

**Keywords:** clozapine, pharmacokinetics, schizophrenia, sertraline, therapeutic drug monitoring

## Abstract

Augmentation of antipsychotic treatment with antidepressants represents a common and beneficial treatment strategy in patients suffering from schizophrenia. Combining clozapine and the selective serotonin reuptake inhibitor (SSRI) sertraline represents a clinically important strategy in patients with therapy‐resistant schizophrenia, but there is limited knowledge about mutual pharmacokinetic interactions. In the present study, we assessed the impact of clozapine on sertraline plasma concentrations. Based on a therapeutic drug monitoring (TDM) database, sertraline plasma concentrations were compared between two groups: patients receiving a combined treatment with sertraline and clozapine (*N* = 15) and a matched control group receiving sertraline but no clozapine (*N* = 17). Group differences with respect to raw and dose‐adjusted concentrations were assessed using nonparametric tests. Comedication with clozapine was associated with 67% lower median sertraline plasma concentrations (16 vs. 48 ng/mL; *p* = .022) and 28% lower median dose‐adjusted plasma concentrations (C/D; 0.21 vs. 0.29 (ng/mL)/(mg/day); *p* = .049) as compared to the control group. Scatter plots revealed a complex relationship between the dosage of clozapine and dose‐adjusted sertraline concentrations composed of an initial decrease at clozapine doses below 300 mg, an increase between 300 and 600 mg and a final decrease at 800 mg which was best modeled by a third order polynomial term. Cotreatment with clozapine may lead to reduced sertraline plasma concentrations which may be explained by clozapine‐induced gastrointestinal hypo‐mobility already present at low doses and cytochrome P450 3A4 inducing properties at high clozapine doses. For this drug combination, clinicians should consider TDM to confirm therapeutically effective plasma concentrations of sertraline.

**Abbreviations**
AGATE: Arbeitsgemeinschaft Arzneimitteltherapie bei psychischen Erkrankungen
CYP: cytochrome P450
SSRI: selective serotonin reuptake inhibitor
TDM: therapeutic drug monitoring

## 1 INTRODUCTION

Growing evidence emphasizes beneficial effects of well‐chosen pharmacological augmentation strategies in the treatment of schizophrenia.^1^, ^2^ In particular, adding antidepressants to antipsychotic monotherapy may alleviate negative symptoms, reduce obsessive‐compulsive behavior, and may improve depressive symptomatology.^3^ Notably, about one‐third of patients suffering from schizophrenia fulfill the criteria of a comorbid major depressive disorder,^4^, ^5^, ^6^ thus significantly exceeding the prevalence of the general population. Accordingly, the concomitant prescription of antidepressant and antipsychotic medication represents a common clinical practice.^7^ Among the different antipsychotic drugs, clozapine is the only antipsychotic with proven efficacy in treatment‐resistant schizophrenia.^8^ The drug is characterized by a pleiotropic receptor profile exerting agonistic effects on the dopamine D_1_‐, serotonin 5‐HT_1A_‐, muscarinic cholinergic M_4_‐ and NMDA‐receptor and antagonistic effects on the dopamine D_2_ ‐ and D_4_‐, serotonin 5‐HT_2A_‐ and 5‐HT_2C_‐, adrenergic α_1_‐ and α_2_‐, histaminergic H_1_‐, muscarinic cholinergic M_1_‐, M_2_‐ and M_3_‐ as well as the GABA_A_‐receptor.^9^ It is extensively metabolized via cytochrome P450 (CYP) isoenzymes mainly involving CYP1A2, 2C19, 2D6, and 3A4^10^, ^11^ and plasma concentrations between 350 and 600 ng/mL are considered to be therapeutically effective.^11^ Selective serotonin reuptake inhibitors (SSRIs) are among the most widely prescribed antidepressant drugs.^12^ Based on meta‐analytical evidence, sertraline is among the most effective antidepressant agents, with a fair tolerability profile.^13^ Sertraline is generally considered to be devoid of clinically relevant CYP‐inducing or inhibiting properties although it exerts weak inhibitory effects on CYP2C19, 2C9, 3A4, and 2D6.^14^, ^15^

Sertraline's metabolism includes N‐demethylation (catalyzed by CYP2B6, CYP2C9, CYP2C19, CYP2D6, and CYP3A4), deamination (catalyzed by CYP2C19, CYP3A4, and monoamine oxidases A and B), and N‐carbamoyl glucuronidation, mediated by UDP‐Glucuronosyltransferase‐2B7.^16^ Sertraline's elimination half‐life is between 22 and 36 hours,^11^ and plasma concentrations in a range between 10 and 150 ng/mL are considered as therapeutically effective.^11^

Only few studies addressed pharmacokinetic or pharmacodynamical interactions between clozapine and sertraline.^17^, ^18^ Specifically, a tendency toward higher plasma concentrations of clozapine in patients comedicated with sertraline compared with clozapine monotherapy has been described in a study controlling for smoking status.^17^ To the best of our knowledge, however, there has been no study addressing the effects of clozapine on sertraline plasma concentrations in patients treated with the combination of clozapine and sertraline. Therefore, we conducted a retrospective analysis of sertraline plasma concentrations from a therapeutic drug monitoring database comparing sertraline concentrations in patients with and without co‐medication with clozapine.

## 2 MATERIALS AND METHODS

KONBEST, a web‐based laboratory information management system for therapeutic drug monitoring laboratories,^19^ served as our data source. The original dataset comprised 1295 sertraline plasma concentrations from 874 patients. Data were collected between 2006 and 2015 as part of the clinical routine in different institutions of the AGATE (Arbeitsgemeinschaft Arzneimitteltherapie bei psychischen Erkrankungen). AGATE represents a collaboration for drug safety in the treatment of mental disorders (for details see: www.amuep‐agate.de). Retrospective analysis of clinical data was in accordance with the local regulatory authority of the medical faculty of the RWTH Aachen University (EK 280/22). In this naturalistic database, patients received treatment with sertraline for different indications and therapeutic drug monitoring was conducted for different reasons such as dosage finding at the beginning of treatment, follow‐up monitoring, change in medication, missing therapeutic effect, estimating the ratio of the concentration and dose, suspected nonadherence or suspected adverse drug effects. Further information, which was available included the date of blood withdrawal, the year of birth, height, weight, gender, an evaluation of the drug concentration according to the dose‐related and therapeutic reference range, the dose of sertraline, names, and doses of simultaneously prescribed drugs, the clinical global impression scale, and smoking and caffeine consumption status. Patients who received a concomitant pharmacological treatment with possible inhibitory or inducing properties for CYP2B6, CYP2C19, CYP3A4, or inhibitory properties for CYP2D6, according to the suggestions by the US Food and Drug Administration,^11^, ^20^ were excluded from the analysis. Among patients with more than one measurement of sertraline plasma concentration, we selected the most recent measurement.^21^ Hence, TDM data of 794 in‐ and outpatients with a broad spectrum of psychiatric diseases were eligible for analysis. Based on this sample, we considered two groups: a group of patients receiving sertraline with co‐medication with clozapine (SERT_CLZ_, *N* = 15, i.e., all patients receiving clozapine and considered eligible according to the above‐mentioned criteria) and a control group receiving sertraline, but no clozapine (SERT, *n* = 17). The control group was extracted out of the remaining 779 patients who did not receive clozapine, by a sequential matching procedure. For each subject of the SERT_CLZ_ group, we first selected all subjects with the same gender. From those subjects, we sequentially selected the subjects with the lowest age difference, lowest weight difference, same status of smoking, same status of caffeine consumption, and lowest dose difference (in this order) as compared to the respective subject of the SERT_CLZ_ group. When no subject of the preliminary control group remained at a certain matching step (e.g., a few subjects were identified having the same gender, the same age, and weight, but all have them had a different smoking status as compared to the subject of the SERT_CLZ_ group used in the current iteration of the algorithm), all subjects of the previous matching step entered the final control group (SERT). Subjects who entered the final control group were not eligible anymore as control subjects for the next subject of the SERT_CLZ_ group (i.e. the next iteration of the algorithm).

Demographic and clinical characteristics of the sample are provided in Table 1. Group differences concerning sociodemographic variables were assessed using Mann–Whitney U‐tests or chi‐squared tests (for the comparison of frequencies).

**TABLE 1:** Sociodemographic and clinical characteristics of the study sample consisting of two groups: patients receiving a cotreatment with sertraline and clozapine (SERT_CLZ_) and a matched control group receiving sertraline, but no clozapine (SERT).

Characteristics	SERT_CLZ_ (*n* = 15)			SERT (*n* = 17)			Comparison	
	Median	Q1	Q3	Median	Q1	Q3	Rank‐sum^a^	*p*
Age [years]	36	24	49	36	25	49	240.0	.790
Dose of sertraline [mg/day]	125	56	150	150	100	150	215.5	.216
Weight [kg]	81	77	120	80	76	101	274.5	.311

	*n*	%	*n*	%	*χ* ^2^ (df = 1)^a^	*p*
Sex					0.03	.863
Female	4	26.7	5	29.4		
Male	11	73.3	12	70.6		
Nicotine consumption					0.43	.513
Smokers	7	46.7	6	35.3		
Nonsmokers	8	53.3	11	64.7		
Caffeine consumption					1.21	.272
Consumers	13	86.7	12	70.6		
Nonconsumers	2	13.3	5	29.4		
Suspected (non‐)adherence					0.08	.78
Suspected nonadherence	6	40.0	6	35.3		
Suspected adherence	9	60.0	11	54.7		

^a^ Group differences in continuous variables were assessed using the Mann–Whitney U‐test (Wilcoxon rank‐sum test), for differences in percentages we applied the chi‐squared test. For the Mann–Whitney U‐test, we report the rank‐sum and the associated *p*‐value.

### 2.1 Quantification of sertraline

Blood samples were drawn just before drug administration (i.e. trough levels) at steady‐state conditions (>5 elimination half‐lives under the same drug dose). All sertraline concentrations were determined in the same laboratory by high‐pressure liquid chromatography with ultraviolet detection (HPLC/UV)^22^; unfortunately, no concentrations of the metabolite desmethylsertraline were available. The method applied here was validated according to DIN 32645 (Deutsche Industrie Norm 32645), described in the guidelines of the GTFCh (Society of Toxicology and Forensic Chemistry) in consideration of ISO 5725 (International Organization for Standardization),^23^ FDA (US Food and Drug Administration) guidance (US Food and Drug Administration, 2018),^24^ and ICH (International Conference on Harmonization) requirements.^25^ The laboratory regularly runs internal quality controls and participates in external quality assessment schemes by INSTAND (Düsseldorf, Germany, www.instandev.de).

Inaccuracy and inter‐ and intraday inaccuracy were evaluated at sertraline concentrations of 300, 100, and 5 ng/mL, respectively.

- Inaccuracy: bias values were—2.14%, 2.80%, and 8.60%
- Interday imprecision: coefficients of variation (CV) were 3.8%, 8.9%, and 13.9%
- Intraday imprecision: CVs were 0.8%, 7.0%, and 10.4%.

Lower limit of detection (LOD) and limit of quantification (LOQ) were 3.6 and 7.2 ng/mL, respectively.

### 2.2 Statistical analysis

Statistical analysis was carried out using MATLAB 2015a (The MathWorks, Inc.) and RStudio (RStudio Team (2020). RStudio: Integrated Development for R. RStudio, PBC, Boston, MA URL http://www.rstudio.com/). Sertraline plasma concentrations were compared between the two groups SERT_CLZ_ (*N* = 15) and SERT (*N* = 17). Dose‐adjusted drug concentrations (ratio of the drug concentration C and the daily dose D, C/D), in [(ng/mL)/(mg/day)] were also calculated. Due to non‐normal distributions of the sertraline concentrations, Mann–Whitney *U*‐test was chosen as a nonparametric test to compare the sertraline plasma concentrations between the two study groups. For the comparison of sociodemographic data, group differences in continuous sociodemographic variables were assessed using the Mann–Whitney *U*‐test (Wilcoxon rank‐sum test), for differences in percentages of categorical variables we applied the chi‐squared test.

In order to evaluate the relationship between the clozapine dose and dose‐adjusted drug concentrations of sertraline, we generated scatter plots and assessed the fit of linear and nonlinear regression models. Among the nonlinear regression models, we tested second and third‐degree polynomials.

### 2.3 Nomenclature of targets and ligands

Key protein targets and ligands in this article are hyperlinked to corresponding entries in http://www.guidetopharmacology.org, the common portal for data from the IUPHAR/BPS Guide to PHARMACOLOGY^26^ and are permanently archived in the Concise Guide to PHARMACOLOGY 2019/20.^27^

## 3 RESULTS

The demographic data of the study groups are displayed in Table 1. No significant differences were found between the groups regarding daily dosage of sertraline, age, weight, sex distribution, caffeine consumption, smoking, or suspected nonadherence (all *p*‐values >.05). Median daily dose of clozapine in the SERT_CLZ_ group was 300 mg (interquartile range [Q1‐Q3] = 150–400 mg).

**FIGURE 1:**
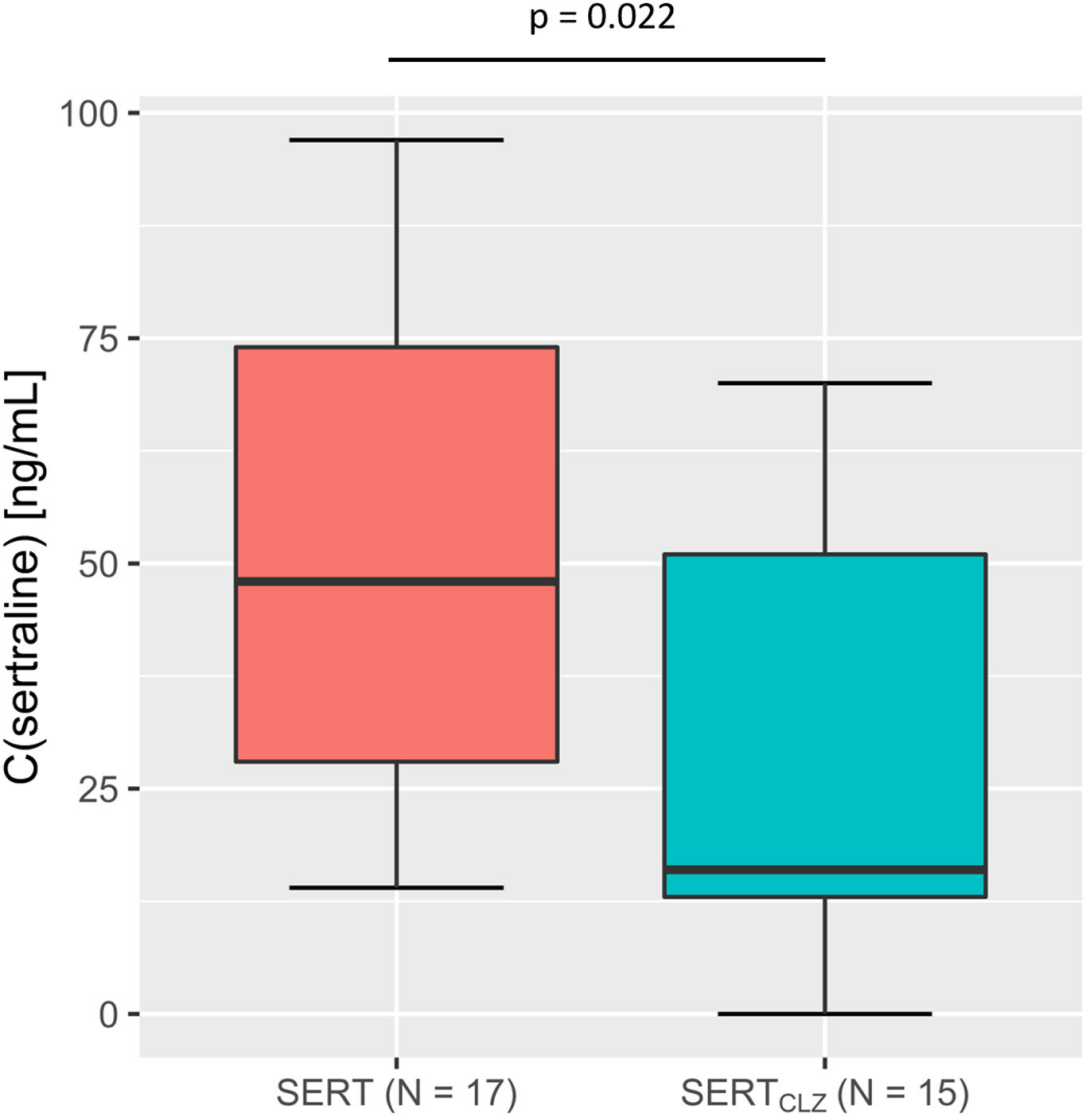
Comparison of sertraline plasma concentrations (ng/mL). Note the significantly lower value in the SERT_CLZ_ group, compared with the control group.

**FIGURE 2:**
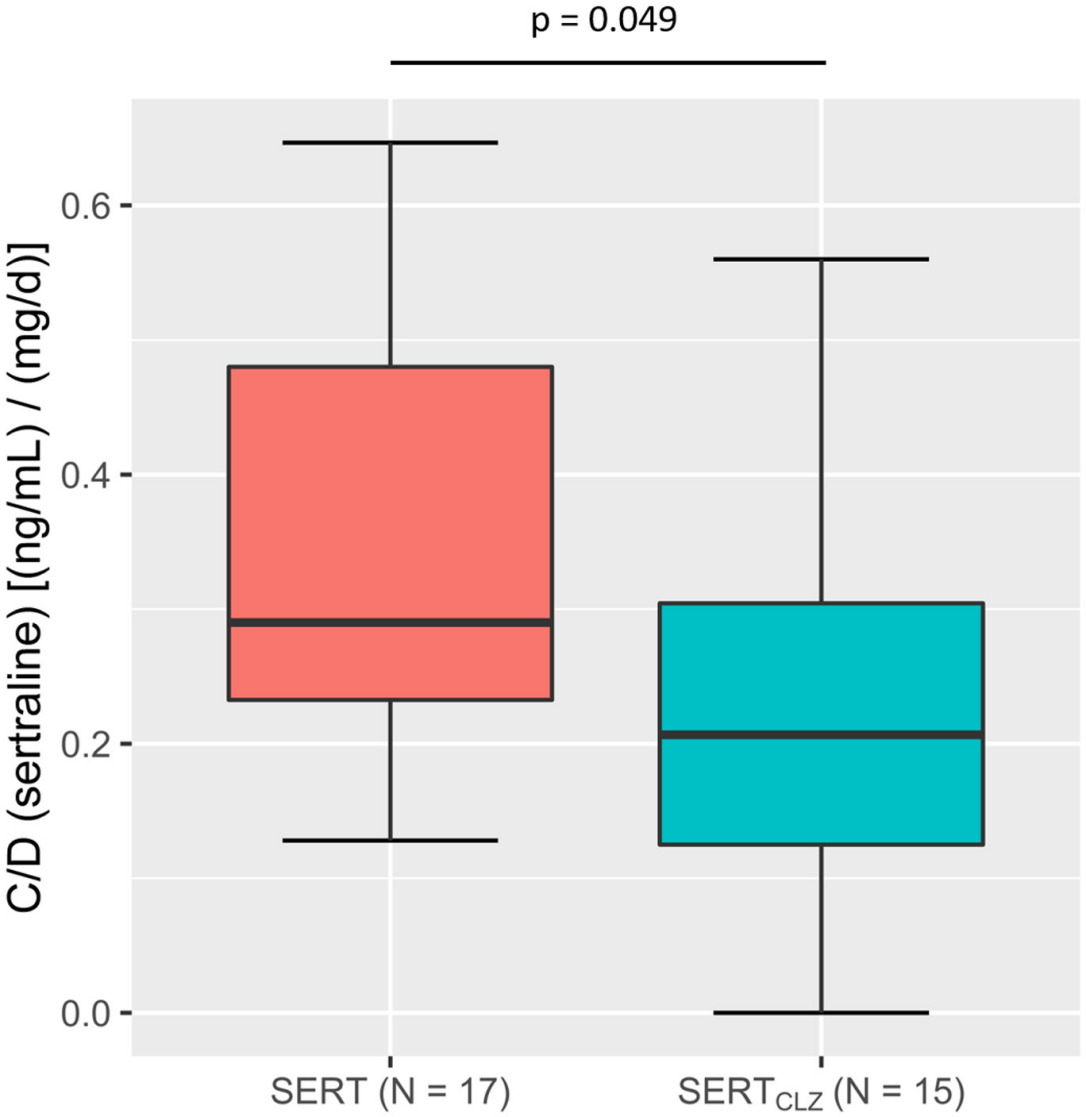
Dose‐adjusted sertraline plasma concentration (C/D) in [(ng/mL)/(mg/day)]. Note the significantly lower C/D value in the SERT_CLZ_ group, compared with the control group.

Plasma concentrations of sertraline were lower in the group comedicated with clozapine compared with the control group (*p* = .022, Mann–Whitney *U*‐test, for detailed statistics see Table 2); accordingly dose‐adjusted drug concentrations were lower in the group comedicated with clozapine compared with the control group (*p* = .049, Mann–Whitney *U*‐test). Specifically, when compared to the control group, patients under a co‐medication with clozapine exhibited 67% lower median plasma concentrations and 28% lower median dose‐adjusted plasma concentrations of sertraline. Moreover, three of the 15 patients in the SERT_CLZ_ group exhibited plasma concentrations below the therapeutic reference range (TRR) of sertraline (10–150 ng/ mL), whereas none of the patients in the SERT group exhibited subtherapeutic concentrations. For a formal comparison of the proportions of patients above and below the lower limit of the TRR between the two groups, we also conducted a chi‐squared test that, however, could only confirm a trend‐level difference in this regard (*χ* ^2^(1) = 3.75; *p* = .053; Figures 1 and 2).

**TABLE 2:** Sertraline plasma concentrations obtained from patients receiving a cotreatment with sertraline and clozapine (SERT_CLZ_) and a matched control group receiving sertraline, but no clozapine (SERT).

Characteristics	SERT_CLZ_ (*n* = 15)			SERT (*n* = 17)			Comparison	
	Median	Q1	Q3	Median	Q1	Q3	Rank‐sum^a^	*p*
Sertraline plasma concentration [ng/mL]	16	13	51	48	27	74.25	186.5	.022
Dose‐adjusted plasma concentrations [(ng/mL)/(mg/day)]	0.21	0.12	0.31	0.29	0.22	0.48	195	.049

^a^ Group differences were assessed using the Mann–Whitney *U*‐test (Wilcoxon rank‐sum test), for which we report the rank‐sum and the associated *p*‐value.

**FIGURE 3:**
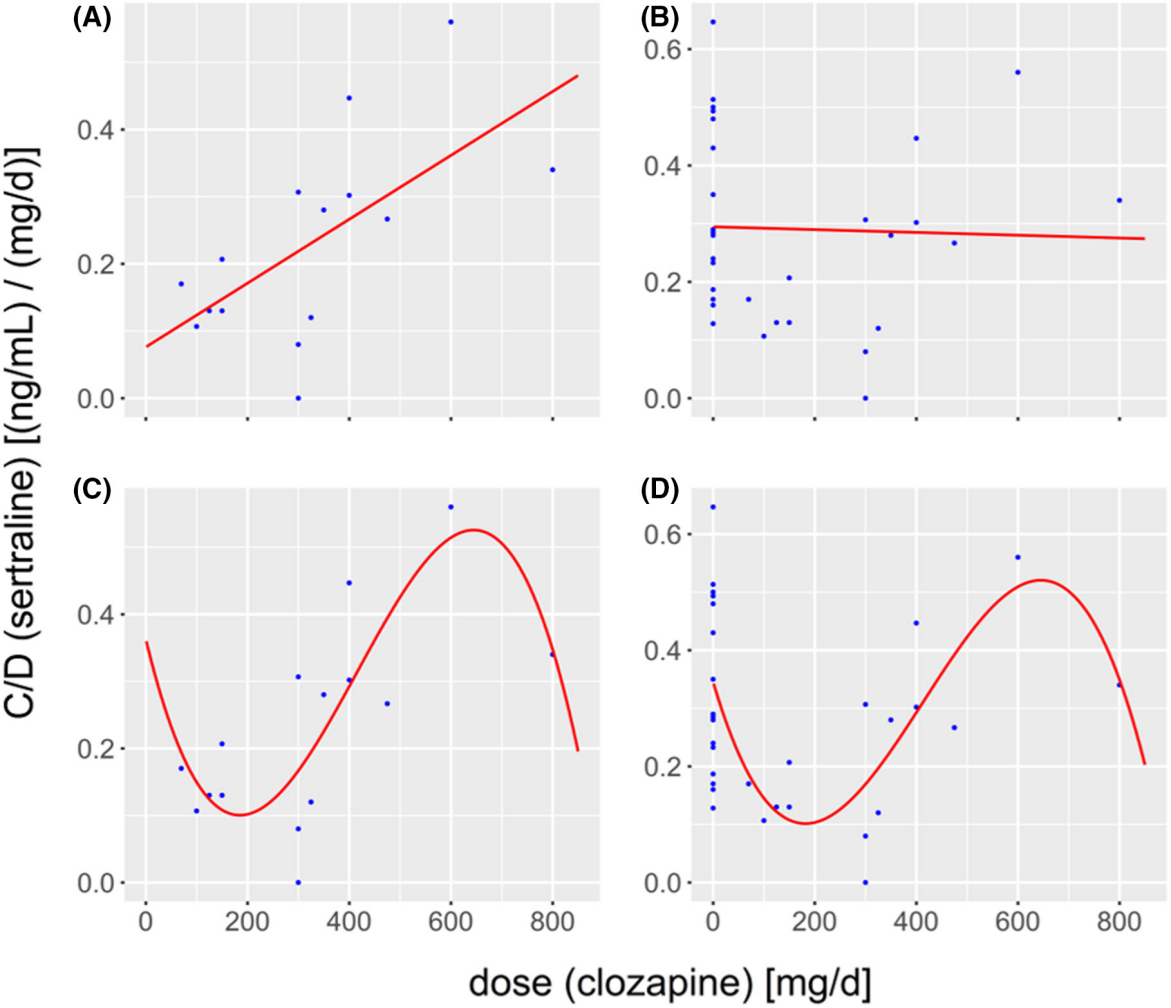
Scatter plots of dose‐adjusted sertraline plasma concentration (C/D) in [(ng/mL)/(mg/day)] versus daily clozapine doses for the SERT_CLZ_ group only (A, C) and the whole sample (SERT + SERT_CLZ_; B and D; note that the SERT group corresponds to a daily clozapine dose of 0 mg). Curves obtained from linear (A, B) and nonlinear (C, D) regression (third‐degree polynomial term) are superimposed on the scatter plots.

Finally, we also assessed the impact of clozapine dose on dose‐adjusted plasma concentrations (C/D) of sertraline, comparing linear and nonlinear regression models. On a descriptive level, scatter plots suggest an initial dip of sertraline concentration for low clozapine doses, followed by an approximately linear increase at clozapine doses between 300 and 600 mg and a final decrease at a clozapine dose of 800 mg (see Figure 3), suggesting that the relationship might be best modeled by a third order polynomial. This last observation (decrease at 800 mg) was, however, only based on one data point obtained at this dose. When restricting the analysis to the SERT_CLZ_ group, only the linear regression model revealed an overall positive relationship (*β* = .0005, SE = 0.0002) and a statistically significant fit (*R* ^2^ = .405; adjusted *R* ^2^ = .359; *F*(1, 13) = 8.84; *p* = .011). While the second‐degree polynomial was associated with a slightly worse, but still significant model fit (*R* ^2^ = .409; adjusted *R* ^2^ = .311; *F*(2, 12) = 4.16; *p* = .043), the third‐degree polynomial exhibited the greatest explanatory power (*R* ^2^ = .618; adjusted *R* ^2^ = .513; *F*(3, 11) = 5.92; *p* = .012). This tendency became more evident when including the SERT group into the analysis (corresponding to a clozapine dose of 0 mg), which revealed that the linear model was not appropriate anymore to explain the data (*R* ^2^ = .001; adjusted *R* ^2^ = −.032; *F*(1, 30) = 0.03; *p* = .863), while the third order polynomial still exhibited the highest explanatory power and was the only model which yielded a statistically significant fit (*R* ^2^ = .373; adjusted *R* ^2^ = .306; *F*(3, 28) = 5.56; *p* = .004, for the complete overview of the statistical details see Table 3).

**TABLE 3:** Statistics obtained from linear and nonlinear regression models assessing the relationship between clozapine dose and dose‐adjusted sertraline plasma concentrations for the SERT_CLZ_ group and the whole sample (SERT_CLZ_ + SERT).

Model	Intercept		Dose (CLZ)		Dose (CLZ)^2^		Dose (CLZ)^3^						
	*β*	SE	*β*	SE	*β*	SE	*β*	SE	*F*	df	*p*	*R* ^2^	Adj. *R* ^2^
SERT_CLZ_													
Linear	0.0761	0.0601	0.0005	0.0002					8.84	1, 13	.011	.405	.359
Poly2	0.2297	0.0319	0.3541	0.1235	−0.0375	0.1235			4.16	2, 12	.043	.409	.311
Poly3	0.2297	0.0268	0.3541	0.1038	−0.0375	0.1038	−0.2539	0.1038	5.92	3, 11	.012	.618	.513
SERT + SERT_CLZ_													
Linear	0.2946	0.0356	−2.4E‐05	0.0001					0.03	1, 30	.863	.001	−.032
Poly2	0.2910	0.0271	−0.0283	0.1532	0.3396	0.1532			2.48	2, 29	.102	.146	.087
Poly3	0.2910	0.0236	−0.0283	0.1335	0.3396	0.1335	−0.4254	0.1335	5.56	3, 28	.004	.373	.306

Abbreviations: Adj., adjusted; CLZ, clozapine; df, degrees of freedom; Poly2/3, second/third‐degree polynomial (note that the linear model is a first‐degree polynomial); SE, standard error.

Since the final decrease in the dose‐adjusted sertraline concentration was only based on one observation (at 800 mg clozapine), we also repeated all regression analyses after removing this data point. This additional set of exploratory analyses confirmed again that a linear model was not the most suitable to explain the data. As to be expected, in this case, a second‐degree polynomial exhibited the best model fit (see Table S1 and Figure S1).

## 4 DISCUSSION

The augmentation of an antipsychotic treatment by adding an antidepressant drug represents a common and potentially beneficial strategy for targeting negative and depressive symptomatology in patients suffering from schizophrenia.^7^, ^28^ The combined treatment with clozapine and sertraline is a clinically particularly meaningful combination due to clozapine's superior potency with respect to treatment‐resistant schizophrenia and sertraline's favorable efficacy and safety profile among different antidepressant drugs. While previous studies focused on the effect of sertraline on plasma concentrations of clozapine, the present study shows—to our knowledge—for the first time that a cotreatment with clozapine and sertraline is associated with significantly lower plasma concentrations of sertraline. In 20% of the co‐medicated patients, plasma concentrations were even below the therapeutic reference range, whereas we did not observe subtherapeutic concentrations in the control group. In accordance with our findings, Srisuma and coworkers^29^ observed elevated plasma concentrations of sertraline associated with a serotonin syndrome 3 days after abrupt discontinuation of clozapine. Elevated drug concentrations may thus be explained by the end of the suppressing effect of clozapine on sertraline plasma concentrations. Subsequent studies are warranted to fully unravel the mechanism of this finding. Indeed, our set of regression analyses assessing the relationship between clozapine dose and dose‐adjusted sertraline concentrations suggests a rather complex situation with probably more than one underlying mechanism.

One potential mechanism might be clozapine's inhibitory effect on gastrointestinal motility, particularly delayed gastric emptying, which is attributed to its antagonistic properties at muscarinic cholinergic, serotonergic, and histaminergic receptors.^30^ Clozapine‐induced gastrointestinal hypomotility typically manifests as constipation, but can also reach life‐threatening stages.^30^, ^31^, ^32^ Importantly, decreasing the rate of gastric emptying leads to a reduced bioavailability of most orally administered drugs.^33^ Since for a majority of drugs, their absorption in the stomach is rather low, a longer dwelling time in the stomach reduces the proportion of unchanged drug available for absorption in the small intestine.^33^, ^34^ Consequently, the here reported lower sertraline plasma concentrations in patients under the concomitant therapy with clozapine may be related to a lower resorption of sertraline due to gastrointestinal hypomotility, particularly delayed gastric emptying. Accordingly, reduced plasma concentrations of SSRIs (including sertraline) have been observed in patients after bariatric surgeries such as the Roux‐en‐Y gastric bypass, which is also associated with delayed gastric emptying.^35^, ^36^ In a considerable amount of patients, decreases in SSRI concentrations after bariatric surgery manifested as SSRI withdrawal syndrome or a relapse of depression. Interestingly, clozapine's negative impact on gastrointestinal motility was also confirmed for low doses of the drug^30^, ^32^ and drug dose did not have an effect on the probability of the occurrence of life‐threatening manifestations.^31^, ^32^ Accordingly, the here reported initial dip of dose‐adjusted sertraline concentrations at rather low doses of clozapine may be related to this mechanism. Another mechanism, which might lead to lower sertraline concentrations in the SERT_CLZ_ group, may be a CYP3A4‐inducing effect of clozapine. Indeed, in a recently published in vitro study using human hepatocytes, Danek et al. provided first evidence for a CYP3A4‐inducing effect of clozapine which was confirmed both for CYP3A4 mRNA and activity level.^37^ However, this effect was only observed for rather high clozapine concentrations starting around the upper limit of the therapeutic reference range. Accordingly, this phenomenon may underlie the second dip of the dose‐adjusted sertraline concentration observed at the clozapine dose of 800 mg in our data. However, this conclusion requires caution as we only collected one observation at this dose. Between these two dips of the sertraline concentration, we observed an increase at clozapine doses between 300 and 600 mg. We assume that this increase is related to the fact that both drugs are substrates of CYP isoenzymes 2C19 and 3A4 and accordingly act as competitive inhibitors.

The present finding may raise the question whether other antidepressant drugs should be preferred over sertraline when considering an augmentation strategy to an antipsychotic treatment with clozapine. In general, SSRIs represent first choice agents due to their favorable properties regarding efficacy and toxicity. Moreover, they exhibit superior efficacy for the treatment of obsessive compulsive symptoms, which are frequently observed in patients with schizophrenia, particularly patients under a treatment with clozapine.^38^, ^39^ Among the different SSRIs, fluoxetine, and paroxetine may increase clozapine levels due to their CYP2D6‐inhibiting properties.^18^, ^40^ Fluvoxamine is a potent CYP1A2 inhibitor and may be used to achieve therapeutically effective plasma concentrations in smokers.^41^ For the newest SSRIs, the racemic compound citalopram (Celexa) and its active S‐enantiomer escitalopram (Lexapro), the cumulative effect on QTc prolongation should be taken into consideration. Since both drugs are metabolized by CYP3A4,^42^, ^43^ the potential CYP3A4‐inducing properties of clozapine might lead to reduced concentrations of both SSRIs, too. Moreover, if our findings are indeed partially explained by a clozapine‐induced decrease in gastrointestinal motility, one would assume similar results for serum concentrations of other SSRIs, antidepressants, and agents belonging to other drug classes as well.

Therefore, sertraline may still be a favorable treatment option for the combination with clozapine, but clinicians may consider the use of therapeutic drug monitoring to confirm the achievement of therapeutically effective plasma concentrations as suggested by the Consensus Guidelines for Therapeutic Drug Monitoring in Neuropsychopharmacology.^11^ However, it has to be noted that the therapeutic reference range of sertraline is very wide and the evidence for the definition of this range is limited. The suggested lower limit of 10 ng/mL is in line with findings obtained from positron emission tomography (PET) studies^44^ which suggested that across different SSRIs, for a therapeutic effect, at least 80% occupancy of striatal serotonin transporters was required. For sertraline, this proportion corresponded to a dose of 50 mg or a plasma concentration of 10 ng/mL, respectively. Our findings may cautiously suggest that due to the higher frequency of subtherapeutic sertraline plasma concentrations in patients co‐medicated with clozapine, the lowest therapeutic dose of 50 mg sertraline may not be therapeutically sufficient for a higher proportion of patients as would be normally expected. If our findings can be reproduced in subsequent and larger studies, clinicians should be aware of this interaction and more frequently consider therapeutic drug monitoring when the symptom domain targeted by sertraline does not respond. Moreover, TDM may determine the individual therapeutic concentration for a given patient.^45^ Once this concentration is known, it may guide dose adjustment when new pharmacokinetic drug–drug interactions are expected to emerge (e.g., clozapine is added to the treatment with sertraline) or pre‐existing interactions are expected to disappear (e.g., clozapine treatment is discontinued, while sertraline treatment is continued).

### 4.1 Limitations

As we retrospectively conducted this analysis, patient information is likely less reliable than in prospective studies. A major limitation of the present study is the limited number of patients. Therefore, the quantitative estimates of clozapine's impact on sertraline plasma concentrations have to be considered as rather uncertain. Another limitation is the non‐normal (right‐skewed) distribution of plasma levels requiring nonparametric statistical tests, which is however, a common finding related to TDM studies (^46^, ^47^). As a further limitation, plasma concentrations of desmethylsertraline, the main metabolite of sertraline, were not available. Moreover, many clinical parameters such as the duration of illness, the clinical phenotype, adverse effects, comorbidities, renal function parameters as well as the duration of prior treatment with sertraline and the co‐medication were not available. In addition, differences in plasma concentrations of sertraline may be also associated with polymorphisms of the corresponding CYP isoenzymes which we, however, did not assess in the present study. Moreover, we cannot definitely exclude the possibility that the findings might be in part explained by differences in adherence. However, as we show in Table 1, the proportion of patients for whom TDM was ordered due to suspected nonadherence did not significantly differ between groups. Therefore, we consider differences in adherence as unlikely to explain the findings obtained in the SERT_CLZ_ group. Finally, it has to be stated that the use of clozapine is generally limited to treatment‐resistant schizophrenia, that is, the here reported findings are only relevant for a subset of patients. However, therapy resistance is highly prevalent in schizophrenia affecting around one third of patients. Nevertheless, clozapine is considered underused in many different regions in the world.^48^, ^49^

## AUTHOR CONTRIBUTIONS

Arnim Johannes Gaebler, Georgios Schoretsanitis, Michael Paulzen, Christoph Hiemke, and Ekkehard Haen participated in research design. Arnim Johannes Gaebler and Michael Paulzen performed data analysis. Arnim Johannes Gaebler, Georgios Schoretsanitis, Katharina Endres, Ekkehard Haen, Christoph Hiemke, and Michael Paulzen wrote or contributed to the writing of the manuscript.

## FUNDING INFORMATION

The research study did not receive funds or support from any source.

## CONFLICTS OF INTEREST STATEMENT

Ekkehard Haen received speaker's or consultancy fees from the following pharmaceutical companies: Servier, Novartis, and Janssen‐Cilag. He is managing director of AGATE, a nonprofit working group to improve drug safety and efficacy in the treatment of psychiatric diseases. He is editor of psiac, an internet based drug–drug interaction program for psychopharmacotherapy (www.psiac.de). He reports no conflict of interest with this publication. Christoph Hiemke has received speaker's fees from Otsuka. He is editor of PSIAC, an internet based drug–drug interaction program for psychopharmacotherapy (www.psiac.de). He reports no conflict of interest with this publication. Michael Paulzen has received speaker's fees from the following pharmaceutical companies: Neuraxpharm, Lundbeck, Janssen, Otsuka. He is editor of psiac, an internet based drug–drug interaction program for psychopharmacotherapy (www.psiac.de). He reports no conflict of interest with this publication. Georgios Schoretsanitis has served as consultant for HLS Therapeutics. All other authors report no conflicts of interests.

## ETHICS STATEMENT

Retrospective analysis of clinical data was in accordance with the local regulatory authority of the medical faculty of the RWTH Aachen University (EK 280/22).

## Supporting information


Table S1

Figure S1

## Data Availability

Data are stored at RWTH Aachen University hospital. The data are not publicly available due to privacy and ethical restrictions.
