# Supplementary material for: Lower sertraline plasma concentration in patients co‐medicated with clozapine—Implications for pharmacological augmentation strategies in schizophrenia
Source: Pharmacol Res Perspect. 2023 Feb 24;11(2):e01065. doi: 10.1002/prp2.1065 (PMC9950877; doi:10.1002/prp2.1065)
Supplement: Supplementary file 1 — Table S1 [file PRP2-11-e01065-s001.docx]

| **Supplementary Table 1.** Statistics obtained from linear and non-linear regression models assessing the relationship between clozapine dose and dose-adjusted sertraline plasma concentrations for the SERT_CLZ_ group as well as the whole sample (SERT_CLZ_ + SERT) after removing a patient receiving 800 mg of clozapine | | | | | | | | | | | | | |
| --- | --- | --- | --- | --- | --- | --- | --- | --- | --- | --- | --- | --- | --- |
|  | | | | | | | | | | | | | |
| SERT_CLZ_ | | | | | | | | | | | | | |
|  | | | | | | | | | | | | | |
|  | intercept | | dose(CLZ) | | dose(CLZ)^2^ | | dose(CLZ)^3^ | |  | | | | |
| Model | beta | SE | beta | SE | beta | SE | beta | SE | F | df | p | R^2^ | Adj. R^2^ |
| Linear | 0.0290 | 0.0670 | 0.0007 | 0.0002 |  |  |  |  | 10.48 | 1, 12 | 0.007 | 0.466 | 0.422 |
| Poly2 | 0.2218 | 0.0277 | 0.3719 | 0.1035 | 0.2013 | 0.1035 |  |  | 8.35 | 2, 11 | 0.006 | 0.603 | 0.531 |
| Poly3 | 0.2218 | 0.0287 | 0.3719 | 0.1075 | 0.2013 | 0.1075 | -0.0469 | 0.1075 | 5.22 | 3, 10 | 0.020 | 0.610 | 0.4932 |
|  | | | | | | | | | | | | | |
| SERT + SERT_CLZ_ | | | | | | | | | | | | | |
|  | | | | | | | | | | | | | |
|  | intercept | | dose(CLZ) | | dose(CLZ)^2^ | | dose(CLZ)^3^ | |  |  |  |  |  |
| Model | beta | SE | beta | SE | beta | SE | beta | SE | F | df | p | R^2^ | Adj. R^2^ |
| Linear | 0.2984 | 0.0369 | -7.0E-05 | 0.0002 |  |  |  |  | 0.17 | 1, 29 | 0.684 | 0.006 | -0.029 |
| Poly2 | 0.2894 | 0.0245 | -0.0678 | 0.1366 | 0.5165 | 0.1366 |  |  | 7.27 | 2, 28 | 0.003 | 0.342 | 0.295 |
| Poly3 | 0.2894 | 0.0244 | -0.0678 | 0.1357 | 0.5165 | 0.1357 | -0.1591 | 0.1357 | 5.37 | 3, 27 | 0.005 | 0.374 | 0.304 |
| CLZ = clozapine; SE = standard error; Poly2 / 3 = second / third degree polynomial (note that the linear model is a first degree polynomial); df = degrees of freedom; Adj. = adjusted. | | | | | | | | | | | | | |
