# Supplementary figures and images for: Lower sertraline plasma concentration in patients co‐medicated with clozapine—Implications for pharmacological augmentation strategies in schizophrenia
Source: Pharmacol Res Perspect. 2023 Feb 24;11(2):e01065. doi: 10.1002/prp2.1065 (PMC9950877; doi:10.1002/prp2.1065)

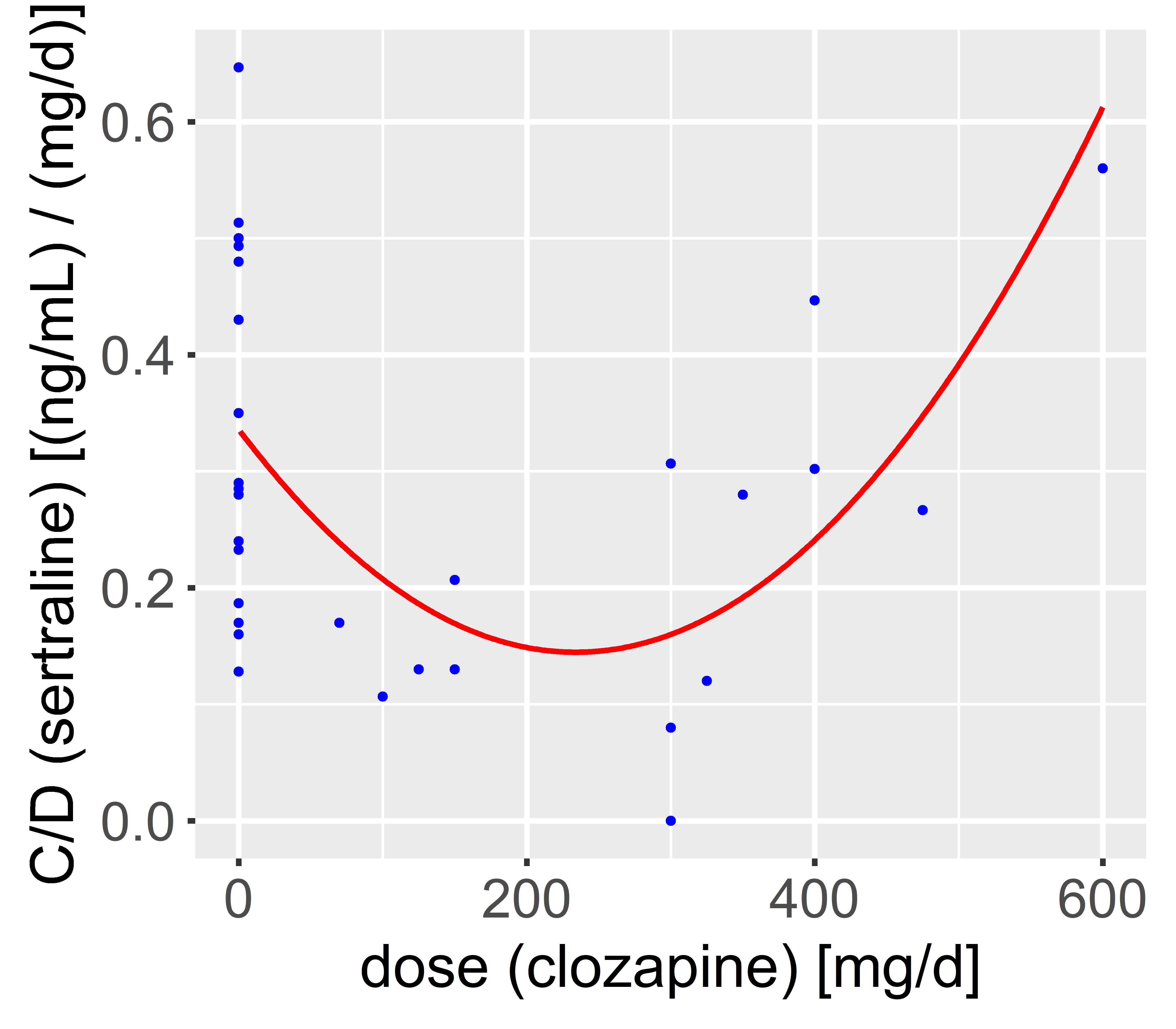

Supplement: Supplementary file 2 — Figure S1 [file PRP2-11-e01065-s002.png]
